# Transcription Factor E2F1 Regulates the Expression of ADRB2

**DOI:** 10.1155/2023/8210685

**Published:** 2023-04-22

**Authors:** Juan Du, Feifei Rui, Zhongfen Hao, Yun Hang, Jin Shu

**Affiliations:** ^1^Department of Pediatrics, The Fourth Affiliated Hospital of Jiangsu University, Zhenjiang, Jiangsu, China; ^2^Department of Neonatology, Changzhou Maternal and Child Health Hospital, Changzhou, Jiangsu, China

## Abstract

Adrenergic beta-2-receptor (ADRB2) is highly expressed in various tissue cells, affecting the susceptibility, development, and drug efficacy of diseases such as bronchial asthma and malignant tumor. However, the transcriptional regulatory mechanism of the human ADRB2 gene remains unclear. This study aimed to clarify whether E2F transcription factor 1 (E2F1) was involved in the transcriptional regulation of the human ADRB2 gene. First, the 5′ flanking region of the human ADRB2 gene was cloned, and its activity was detected using A549 and BEAS-2B cells. Second, it was found that the overexpression of E2F1 could increase promoter activity by a dual-luciferase reporter gene assay. In contrast, treatment of knockdown of E2F1 significantly resulted in a decrease in its promoter activity. Moreover, mutation of the binding site of E2F1 greatly reduced the potential of human ADRB2 promoter transcriptional activity to be regulated by E2F1 overexpression and knockdown. Additionally, by real-time quantitative PCR and Western blot analysis, we demonstrated that overexpression of E2F1 elevated the ADRB2 mRNA expression and protein levels while si-E2F1 reduced its expression. Finally, the consequence of the chromatin immunoprecipitation assay showed that E2F1 was able to bind to the promoter region of ADRB2 in vivo. These results confirmed that E2F1 upregulated the expression of the human ADRB2 gene.

## 1. Introduction

ADRB2 gene is a 1200 bp intron-free gene located in the 5q31-q32 asthma susceptibility fragment [1, 2], encoding beta-2-adrenergic receptor (*β*2-AR), a member of the G protein-coupled transmembrane receptor superfamily, which can specifically bind catecholamine and other endogenous ligands and be activated by their agonists to produce a series of biological effects [3, 4].

ADRB2 is highly expressed in bronchial smooth muscle and has been proven to play an important role in the development of asthma. It mediated airway physiological activities such as bronchodilation, ciliated mucilage clearance [5, 6], inhibition of cholinergic nerve action [7], suppression of microexudation [8], and inhibition of mast cells and basophil releasing mediators [9, 10]. A growing number of studies have shown that the ADRB2 gene influenced the susceptibility to asthma severity, and the *β*2-AR agonist correlation of response. A meta-analysis demonstrated that ADRB2 haplotypes functioned as defensive factors for asthma [11]. Fu et al. [12] observed the dose-dependent relationship between ADRB2 5′-UTR methylation and risk for severe asthma. Recent evidence has shown that ADRB2 expression in patients with asthma is positively correlated with FEV1 and the response of salmeterol to asthma [13, 14]. In addition, *β*2-AR is highly expressed in most malignancies. Mounting evidence shows that activation of *β*2-AR signal pathway promotes the carceration, aggression, and metastasis of malignancies, which is related to the progression and treatment resistance of various malignant tumors, including breast cancer, gastric cancer, prostate cancer, and pancreatic cancer [15–19]. Moreover, recent research has shown that catecholamine significantly increased the proliferation, invasion, and viability of tumor cells in vivo, and this effect could be inhibited by an ADRB2 antagonist through suppression of the ERK1/2-JNK-MAPK pathway and transcription factors [20].

The E2F transcription factors (E2Fs) family is an important transcription factor family that regulates gene expression, which was discovered in 1986 by Kovesdi et al. [21]. Numerous studies have suggested that E2F1 can influence gene expression through the regulation of transcription and the half-life of RNA molecules [22, 23]. In fact, advances in high-throughput techniques have shown that E2F1 can bind to the promoters of a significant number of genes involved in most cellular pathways, influencing gene transcription and expression [24]. It was demonstrated that E2F1 regulated the transcriptional levels of target genes depending on the ERK1/2 pathway in advanced non-small cell lung cancer (NSCLC) [25]. Nevertheless, little is known about the transcriptional regulatory effects of E2F1 on the human ADRB2 gene.

In the present study, considering that ADRB2 is an asthma susceptibility gene and E2F1 is closely related to the occurrence of lung cancer and other diseases, we performed a dual-luciferase reporter gene assay in two different cell lines—A549 (human pulmonary adenocarcinoma cell) and BEAS-2B (transformed human bronchiolar epithelial cell), verifying that the transcription factor E2F1 served as a transcriptional activator of the human ADRB2 gene by binding to its promoter. Further, our results demonstrated that E2F1 increased the expression both mRNA expression and protein levels of the human ADRB2 gene. To the best of our knowledge, this study provides the first experimental evidence for the involvement of E2F1 in the transcriptional regulation of the ADRB2 gene.

## 2. Materials and Methods

### 2.1. Cell Lines and Culture

The A549 and BEAS-2B cell lines used here were purchased from the American Type Culture Collection. All cell lines were cultured at 37°C with 5% CO_2_ in RPMI Medium 1640 (Gibco) with 10% fetal bovine serum (Gibco), 100 units/ml penicillin, 100 *μ*g/ml streptomycin, and 100% humidity.

### 2.2. Plasmid Construction and Small Interfering RNA (siRNA)

Based on the ADRB2 gene (ENSG00000169252) sequences reported in the Ensembl database, we can find the sequence (−1,879 to 39) upstream of the transcription start site (TSS). The ADRB2 promoter fragment was obtained by polymerase chain reaction (PCR) using primers containing KpnI and HindIII restriction sites, which were designed with Primer 5.0 and synthesized by Sangon (Shanghai, China). The primer sequences were as follows: sense: 5′-CGGGGTACCCTCAGGCAGACCTGGGTCAAATCC-3′, and antisense: 5′-CCCAAGCTTAGTTCCAGCCCGTGCTCTGAAGAA-3′. The products of PCR and the luciferase reporter plasmid pGL3-basic (Invitrogen) were respectively digested using KpnI and HindIII, and then recombined them using T4 DNA ligase (Thermo). The products were used to transform *E. coli* DH5*α* competent cells, verified by Sanger sequencing and the resulting plasmid was termed pGL-1879/+39. According to the JASPAR (https://jaspar.genereg.net/), a series of the potential transcriptional binding sites of E2F1 were predicted (E2F1a-e). Moreover, according to the critical regulatory regions of the human ADRB2 gene promoter (−219∼−1, Kobilka et al. [26]), the bioinformatics tools UCSC Genome Browser were used to evaluate the phylogenetic conservation of E2F1 binding sites in this section (E2F1Dd, E2F1e). Then, the binding site E2F1e (−123∼−112), which was closest one to the TSS, was identified and suffered from mutation. The mutant plasmid named mut-E2F1 was synthesized based on the previous cloning of pGL-1879/+39. The mutated sequence of the E2F1 binding site (−123 GGGGAGGGAAA−112) was 5′-GGGGCAAGAAA-3′. Knockdown of the E2F1 was achieved by transfecting siRNA into A549 and BEAS-2B cells, respectively. The siRNA of E2F1 (si-E2F1) and the negative control (si-NC) were synthesized by Sangon (Shanghai, China). The following primers sequences (Stoleriu et al. [27]) were utilized in the research: sense: 5′-GACGUGUCAGGACCUUCGU-3′, and antisense: 5′-ACGAAGGUCCUGACACGUC-3′. The overexpression plasmid pcDNA-E2F1 and its corresponding negative control plasmid pcDNA3.1 were kept by our laboratory [28].

### 2.3. Transient Transfection and Dual-Luciferase Assay

Transient transfection of A549 and BEAS-2B cells was performed using Lipofectamine™ 3000 (L3000015, Invitrogen), according to the manufacturer's instructions. We used the RPMI Medium 1640 with 10% fetal bovine serum without any antibiotic to improve the effectiveness of transfection. The cells were seeded in 96-well plates (1.5 × 10^4^/well) and cotransfected after 24 h with 100 ng of the promoter reporter plasmids (pGL3-basic, pGL-1879/+39, and mut-E2F1) together with the pRL-TK plasmid (used as an internal control; 4 ng; Promega). After another 24 h, the cells were harvested and assayed to detect the relative luciferase activities by the dual-luciferase reporter assay system (Promega). For overexpression or knockdown of E2F1 experiments, an additional 100 ng of pcDNA3.1, pcDNA-E2F1 or 100 nM of si-E2F1, si-NC (supplemental material for the experiments to verify that these were able to change the E2F1 protein levels as expected) were added to the transfection system as described above and cotransfected into cells with those reporter plasmids, respectively. At 24 h post-transfection, cells were lysed, and the luciferase activities were measured. All the experiments were performed independently in triplicate.

### 2.4. Quantitative Real-Time PCR (qRT-PCR)

For qRT-PCR, briefly, total RNA was isolated with TRIzol reagent (Invitrogen, USA) according to the manufacturer's instructions and then reverse-transcription was performed using HiScript III Reverse Transcriptase (Vazyme Biotech Co., Ltd.) to obtain cDNA. qPCR amplification was conducted using SYBR Green I Master Mix (Vazyme Biotech Co., Ltd.) with GAPDH being used as an internal control. The following primers of ADRB2 synthesized by Sangon (Shanghai, China) were as follows: sense: 5′-GTGATCATGGTCTTCGTCTACT-3′, antisense: 5′-CATGATGATGCCTAACGTCTTG-3′. Each reaction was performed in triplicate.

### 2.5. Western Blot Analysis

Total proteins of ADRB2 and E2F1 were extracted, respectively, in accordance with the instructions of the RIPA buffer (Solarbio) with 5x sample loading buffer (Beyotime) added and boiled at 100°C for 5 minutes. 10% SDS-PAGE gel was made using the SDS-PAGE Gel Kit (Solarbio), 75 V pre-electrophoresis, and 120 V constant pressure separation. The protein was transferred to PVDF membrane at a constant flow rate of 250 mA after 120 minutes at room temperature. After blocking with 5% skim milk powder in TBST saline (0.25 M Tris-HCl, 0.19 M NaCl and 0.1% Tween 20) solution for 2 h, the primary antibody anti-ADRB2 (1 : 2000; Abcam; ab182136), anti-E2F1 (1 : 2000; Abcam; ab288369) and GAPDH Mouse McAb (1 : 2000; ProteinTech; cat. no. 60004-14-1-Ig) were utilized to treat the protein blots at 4°C overnight. Subsequently, the secondary antibody was incubated with anti-Rabbit IgG HRP (1 : 2000; Biosharp; BL003A) or HRP-conjugated goat anti-mouse IgG (1 : 2,000; ProteinTech; cat. no. SA00001-1) at room temperature for 2 h after washing with TBST. Next, the bands were visualized with the ECL reagent (L/N 7E410L0, Vazyme). Blot images were captured under the ChemiDoc XRS Image Lab System (1708265, BioRad) and further analyzed utilizing the Image J software. GAPDH were used as controls and were detected with mouse monoclonal anti-GAPDH antibody (Santa Cruz, CA). The experiment was repeated three times independently.

### 2.6. Chromatin Immunoprecipitation Assay (ChIP)

The ChIP assay was performed using the EZ-Magna ChIP A-Chromatin IP Kit (17–408, Millipore). BEAS-2B cells were cross-linked with 1% formaldehyde and stopped by the addition of 0.125 M glycine. The cells were cleaned in ice-cold PBS, scraped off, and collected into a 15 ml centrifuge tube. Protease inhibitor complexes were added to the cells lysed in cell lysis buffer. Sonication of cross-linked chromatin was performed at 200 watt with 30 rounds of 5 seconds pulses. The chromatin fragments were 150–200 bp in size, as verified by agarose gel electrophoresis. Diluted chromatin (1%) was collected and used as input. Subsequently, anti-E2F1 (3742S, CST), anti-acetyl histone H3 (positive control), and normal anti-Rabbit IgG (negative control) were added and incubated overnight at 4°C for immunoprecipitating the chromatin. Protein/antibody/chromatin complexes were pelleted through the magnetic beads. Beads were washed four times with ice-cold wash buffer (Low Salt Immune Complex Wash Buffer, High Salt Immune Complex Wash Baffer, Licl Immune Complex Wash, and TE Buffer). When the complexes were eluted by resuspension of the washed pellet in 1 M NaHCO_3_ and 1% SDS for 30 minutes, proteinase K was added into and incubated at 62°C for 2°h and 95°C for 10 minutes to obtain separate DNA. The samples were then purified using mini-column centrifugation. The purified DNA was amplified by PCR and separated by gel electrophoresis on a 1% agarose gel. Enrichment of the DNA fragment was detected by qRT-PCR with SYBR Green I Master Mix (Vazyme). The specific primers used were as follows: sense: 5′-GGACACCACCTCCAGCTTTA-3′, antisense: 5′-GTGACGTACGGGAACTTTCG-3′.

### 2.7. Statistical Analysis

The results are expressed as the mean ± standard error of the mean. Statistical analysis was performed using unpaired t-test with SPSS software (version 20.0; SPSS, Inc., Chicago, IL, USA). *P* < 0.05 was considered to indicate a statistically significant difference.

## 3. Result

### 3.1. The Predicted Promoter Fragment of Human ADRB2 Is Functional in A549 and BEAS-2B Cell Lines

**Figure 1:**
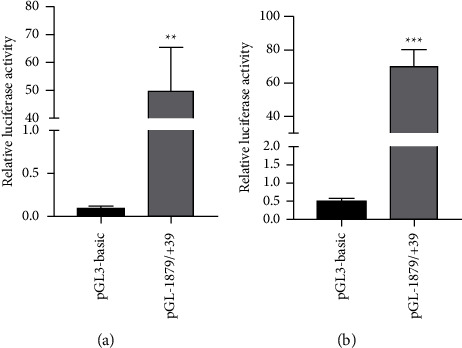
Construction and identification of the human ADRB2 promoter. A549 and BEAS-2B cells were seeded in 96-well plates, followed by transfection with pGL-1879/+39 and pGL3-basic. After 24 h, the relative luciferase activities were detected. Compared to the pGL3-basic, the activity of pGL-1879/+39 was markedly enhanced in A549 ((a) ^*∗∗*^*P* < 0.01) and BEAS-2B ((b) ^*∗∗∗*^*P* < 0.001). All the experiments were performed independently in triplicate.

After DNA sequencing verification, the luciferase assay was performed to determine the functionality of pGL-1879/+39 in A549 and BEAS-2B cells. The activity of pGL-1879/+39 was 491 and 140 times higher than that of the control plasmid pGL3-basic, suggesting that the recombinant plasmid was successfully constructed (Figures 1(a) and 1(b)).

### 3.2. E2F1 Is a Transcriptional Activator of the Human ADRB2 Gene

**Figure 2:**
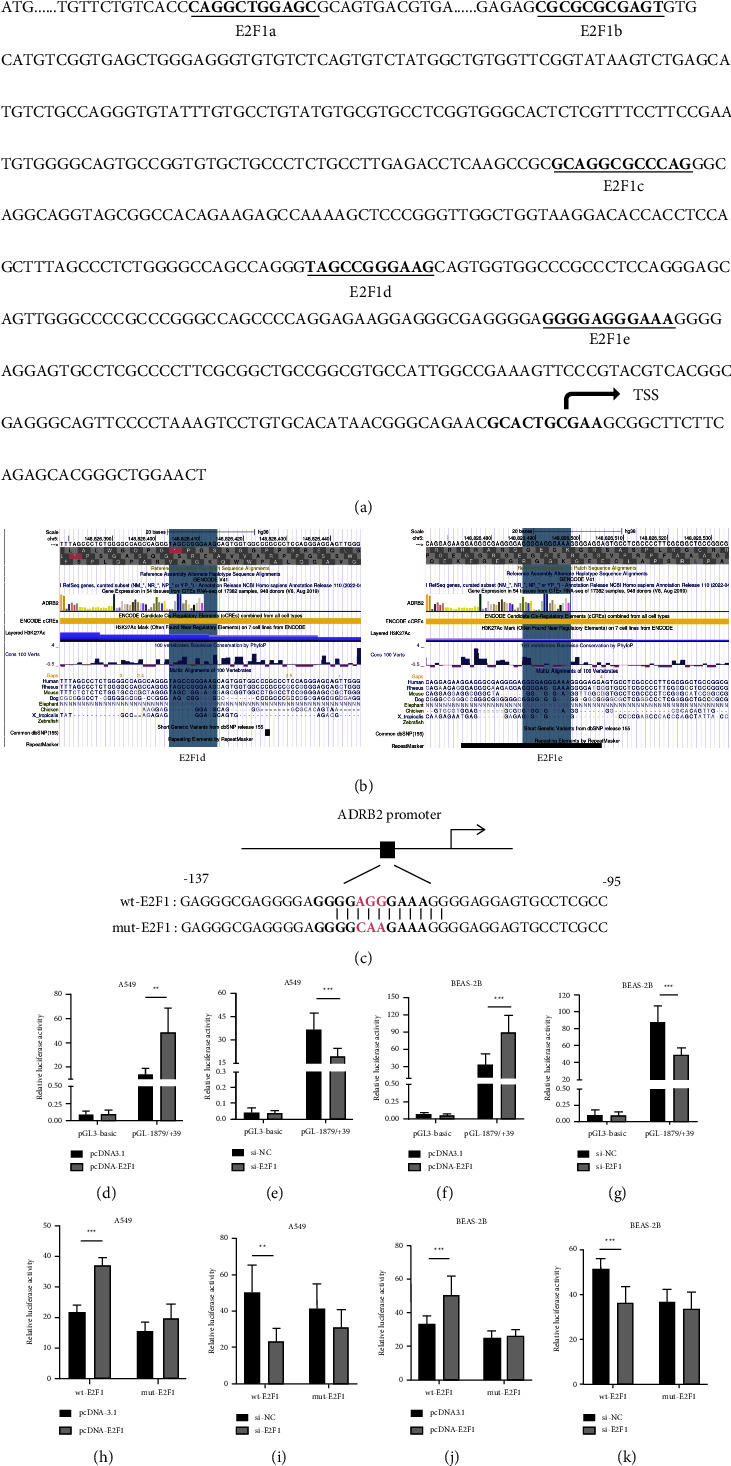
(a) Prediction of binding sites of E2F1 using JASPAR. (b) Phylogenetic conservation analysis of the E2F1 binding sites on human ADRB2 gene promoter. (c) The mutation of the putative binding site E2F1 on the human ADRB2 promoter around 130 bp. (d–g) Regulation of ADRB2 promoter activity by E2F1 in A549 and BEAS-2B cells. The change of E2F1 level did not affect the activity of the pGL3-basic empty vector. The pGL3-basic or pGL-1879/+39 plasmids were cotransfected with pcDNA3.1 (100 ng) or pcDNA-E2F1 (100 ng) into A549 (d) and BEAS-2B (f) cells. Luciferase activity was detected 24 h following transfection (^*∗∗*^*P* < 0.01, ^*∗∗∗*^*P* < 0.001). At the same condition, the pGL3-basic or pGL-1879/+39 plasmids were cotransfected with si-NC (100 nM) or si-E2F1 (100 nM) into A549 (e) and BEAS-2B (g). Luciferase activity was detected 24 h following transfection (^*∗∗∗*^*P* < 0.001). (h–k) Equal amounts of reporter plasmids of human ADRB2 promoter containing wild or mutant E2F1 sites were cotransfected with plasmids of E2F1 overexpression or knockdown into A549 ((h) ^*∗∗∗*^*P* < 0.001, (i) ^*∗∗*^*P* < 0.01) and BEAS-2B ((g) ^*∗∗∗*^*P* < 0.001, (k) ^*∗∗∗*^*P* < 0.001) cells. The relative luciferase activities were detected.

The presumptive transcription factor binding sites of E2F1 on the human ADRB2 gene promoter were predicted using the bioinformatics tool JASPAR (Figure 2(a)), indicating that E2F1 might have a potential function to regulate the expression of human ADRB2 gene. At the same time, phylogenetic conservation analysis was performed for the −230 to −1 sequences in the upstream TSS of the human ADRB2 gene. The results showed that the E2F1 binding sites in this sequence were subjected to partial variation in different species (Figure 2(b)). To investigate whether E2F1 regulated the activity of the ADRB2 promoter, the overexpression/knockdown of E2F1 plasmids and the negative control were respectively cotransfected into A549 and BEAS-2B cells. Then, the activities of the pGL3-basic empty vector with and without E2F1 overexpression/knockdown were measured to make sure that the empty vector does not respond to changes in E2F1 levels (the activity of the pGL3-basic empty vector in different samples was close to zero with no significant difference, as shown in Supplemental files Figure 1). As shown in Figures 2(d) and 2(f), the overexpression of E2F1 significantly increased the luciferase activity of pGL-1879/+39 in A549 and BEAS-2B cells by 2.46 times and 1.73 times, respectively. On the contrary, knockdown of E2F1 led to a remarkable decrease in the promoter activity by 45.7% and 43.3% in A549 and BEAS-2B cells, respectively (Figures 2(e) and 2(g)). Moreover, mutation of the putative binding site E2F1e (GGGGAGGGAAA, Figure 2(c)) made it lose its potential to regulate the transcriptional activity of the human ADRB2 gene promoter by E2F1 overexpression and knockdown (Figures 2(h)–2(k)). These results indicated that E2F1 may play a role of positive regulation in the transcription of the promoter of the human ADRB2 gene by binding to the site predicted.

### 3.3. E2F1 Binds to the Promoter of Human ADRB2 Gene In Vivo

**Figure 3:**
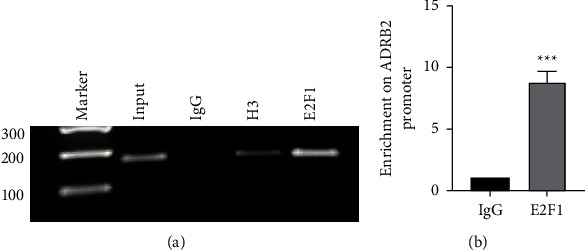
E2F1 binds to the promoter of human ADRB2 gene in vivo. ChIP assay was performed in untreated BEAS-2B cells to confirm the binding of E2F1 to human ADRB2 promoter. The cross-linked DNA-protein complexes were incubated with anti-E2F1 and separated by immunoprecipitation. ChIP products were amplified by PCR reaction. (a) There were signal beneficiation on input, anti-histone H3 antibody and anti-E2F1 antibody, whereas no signal was observed on anti-IgG using agarose gel electrophoresis. (b) Relative enrichment of E2F1 on the promoter region of ADRB2 was assayed by qRT-PCR (^*∗∗∗*^*P* < 0.001).

In the previous experiments, we speculated that E2F1 could regulate the ADRB2 promoter activity by binding to the upstream sequence of TSS. Therefore, chromatin immunoprecipitation assay was performed in untreated BEAS-2B cells to validate whether E2F1 interacted with the ADRB2 promoter in vivo. As shown in Figures 3(a) and 3(b), an enrichment of the ADRB2 promoter was monitored using anti-E2F1 antibody in BEAS-2B cells, which suggested that E2F1 was able to bind to the promoter region of human ADRB2 gene in vivo.

### 3.4. E2F1 Upregulates the Expression of Human ADRB2 Gene

**Figure 4:**
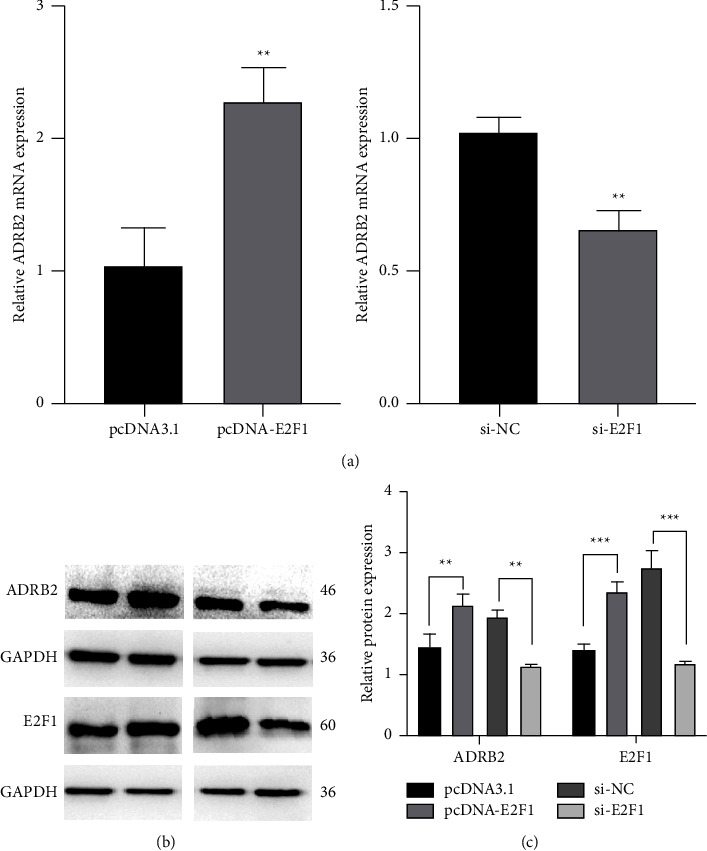
E2F1 upregulates the expression of human ADRB2 gene. (a) BEAS-2B cells were seeded in 12-well plates at a concentration of 1.5 × 105 cells per well 24 h before pcDNA-E2F1 or si-E2F1. After 24 h, cells were harvested for RNA, and reverse-transcription was performed to obtain cDNA for qRT-PCR. GAPDH was utilized as a housekeeping gene for calibration (^*∗∗*^*P* < 0.01). BEAS-2B cells were seeded in 6-well plates at a concentration of 3.0 × 10^5^ cells per well and pretreated with the same condition, as described above. Forty-eight hours after transfection, cells were harvested and subjected to Western blot analysis. Equivalent amounts of total cell proteins were fractionated by SDS-PAGE and probed with antibodies specific for ADRB2, E2F1, and GAPDH. The band was visualized with the ECL reagent (b) and further analyzed utilizing Image J (c, ^*∗∗*^*P* < 0.01, ^*∗∗∗*^*P* < 0.001).

In order to further examine the effect of E2F1 on the expression of human ADRB2 gene, the mRNA expression and protein levels assay of ADRB2 using qRT-PCR and Western blotting in BEAS-2B cells were carried out. Meanwhile, Western blotting was used to verify the efficiency of E2F1 overexpression and knockdown. BEAS-2B cells were transfected with siRNA or E2F1-overexpression plasmid. Then qRT-PCR using an equal amount of RNA was performed with specific primers to ADRB2 and GAPDH (control). Compared with the control vector, the overexpression of E2F1 enhanced the mRNA expression by 1.23 times. Oppositely, si-E2F1 resulted in downregulation of its expression by 36% (Figures 4(a) and 4(b)). Correspondingly, cells were treated with the same condition as described previously. After 48 h, the lysates were obtained, and Western blotting was performed to analyze the expression of E2F1, ADRB2 and GAPDH in the same sample. As shown in Figures 4(c), overexpression of E2F1 could effectively improve the protein levels of ADRB2 and E2F1 by 46% and 67.6%, respectively. In contrast, knockdown E2F1 was able to efficaciously reduce its levels by 42.1% and 58%. In addition, Western blotting analyses were performed on the lysates extracted from BEAS-2B cells transfected with equivalent amounts of ADRB2 and E2F1, and the result showed that there was no statistical significance between their control group, which further proved that the change in E2F1 was able to regulate the expression of ADRB2. These results suggested that E2F1 could positively regulate the expression of the human ADRB2 gene at mRNA and protein levels.

## 4. Discussion

The present study focused on the basal transcription regulation of the human ADRB2 promoter. Jiang and Kunos [29] demonstrated that transcriptional enhancement of the rat ADRB2 promoter may be mediated by the element resided in the 500 bp fragment. Jaeger et al. [30], constructing a stepwise deletion of the 5′ flanking sequence of the promoter region (−1324/+33∼−269/+33), revealed that the minimal promoter of porcine ADRB2 was located in the −307∼−209 region. Compared with the pGL3-basic reporter vector, promoter activity of pGL-307/+33 was 3-fold increased in COS-7, 7-fold increased in HepG2, 5-fold increased in C2C12, and 2-fold increased in 3T3-L1. Also, this study has identified the importance of −400∼−209 region in the regulation of basal expression of porcine ADRB2 and the lack of the presence of key regulatory element in the 5′ flanking −882∼−709 region. Johnatty et al. [31] showed that the majority of promoter activity resides within a 549 bp fragment immediately 5′ to the start of translation and identified four naturally occurring polymorphisms (−468C ⟶ G, −367T ⟶ C, −47T ⟶ C, −20T ⟶ C). These variants led to highly significant alteration of the luciferase activity of the human ADRB2 promoter. The pCTTC and pCCTC constructs led to an increase in promoter expression of 30% compared with the normalized construct pCTTT. Two other constructs, pCTCT and pGCCT showed reduction of three times. In our present study, a 1918 bp fragment of the human ADRB2 promoter was cloned into a reporter plasmid and a dual-luciferase reporter gene assay was performed. The luciferase assayed revealed a 491-fold increase in ADRB2 promoter activity in A549 and a 140-fold increase in BEAS-2B from pGL-1879/+39 compared with the empty pGL3-basic reporter vector, indicating a functional promoter in the pGL-1879/+39 region of ADRB2.

E2F1 is generally considered to be “activating E2F” and mainly binds the retinoblastoma protein (pRB) in a cycle-dependent manner, that is, regulates cell G1/S process through the nucleus RB/E2F pathway [32, 33]. It is supposed to relate to the occurrence and development of proliferative diseases. Compared with normal bronchial epithelial cells, E2F1 expression in the lung tissues of asthma patients was different and regulated by c-Myc that regulated cell proliferation and apoptosis [34]. The latest research showed that E2F1 affected TGF-*β*1-induced pulmonary fibrosis and epithelial-mesenchymal transition (EMT) in BEAS-2B cells through the miR-106b-5p/E2F1/SIX1 signaling pathway [35]. In addition, mutations in retinoblastoma tumor suppressor genes (RB1) or components that regulate the CDK/RB/E2F pathway are present in almost every human malignancy. In breast cancer, there is often an EMT associated with RB-E2F1 pathway [36]. Meng et al. [37] illustrated that the proliferation, migration, and EMT of non-small cell lung cancer (NSCLC) cells were promoted through E2F1 bound to the promoter of long intergenic nonprotein coding RNA 461 (LINC00461), a molecule with oncogenic potential in several cancers. Moreover, overexpression of E2F1 has been reported to be related to tumor growth and cell survival in prostate cancer (PCa), whereas knockdown treatment of E2F1 inhibited cell cycle progression, invasion, and migration of PCa cell lines in vitro, as well as tumor growth and EMT in vivo [38].

Previous studies have shown that EZH2 was able to bind to the human ADRB2 promoter and repressed ADRB2 expression, which promoted metastatic prostate cancer [39]. Boulay et al. [40] have confirmed that ADRB2 is a new direct target gene of HIC1, an inhibitor for tumor invasion. Treatment of HIC1 with small interference was able to downregulate the expression of ADRB2, thereby reducing the migration and invasion of MDA-MB-231 cells which are highly malignant breast cancer cells. In present study, we found that human ADRB2 promoter contained several E2F1 binding sites by bioinformatics analyses. Overexpression of E2F1 enhanced the promoter activity of ADRB2, on the contrary, knockdown of E2F1 using siRNA decreased its activity. The mutation of an E2F1 putative binding site in the key regulatory region of the ADRB2 promoter revealed that it lost its potential to regulate the transcriptional activity of the human ADRB2 gene promoter, although this site was not conserved consistently in different species. We also demonstrated that E2F1 was able to bind to the promoter of ADRB2 in vivo by the ChIP experiment. Moreover, overexpression of E2F1 upregulated both mRNA expression and protein levels of the human ADRB2 gene, as well as knockdown of E2F1 led to a significant reduction in its expression. Accordingly, we speculate that E2F1 may affect the progression and prognosis of various diseases such as asthma, malignant tumor through basal transcriptional regulation of human ADRB2 gene, and that E2F1 may become a new therapeutic target and prognostic marker. But the specific regulation mechanism still needs further study.

To sum up, in this study, we confirmed that E2F1 was able to bind to the ADRB2 promoter in vivo. Meanwhile, E2F1 can upregulate the promoter activity and expression of ADRB2 at the basal transcriptional level. These results may provide novel ideas for further understanding the molecular regulatory mechanism of the human ADRB2 gene.

## Data Availability

The data used to support the findings of this study are available from the corresponding author upon request.

## Supplementary Materials

Supplementary Materials Accompanies this study at supplemental file 1, which was the correlation analysis of luciferase activity of pGL3-basic in Figures 2(d)–2(h).
