# Supplementary material for: Transcription Factor E2F1 Regulates the Expression of ADRB2
Source: Int J Anal Chem. 2023 Apr 22;2023:8210685. doi: 10.1155/2023/8210685 (PMC10148742; doi:10.1155/2023/8210685)
Supplement: Supplementary Materials — Accompanies this study at supplemental file 1, which was the correlation analysis of luciferase activity of pGL3-basic in Figures 2(d)–2(h). [file 8210685.f1.pdf]

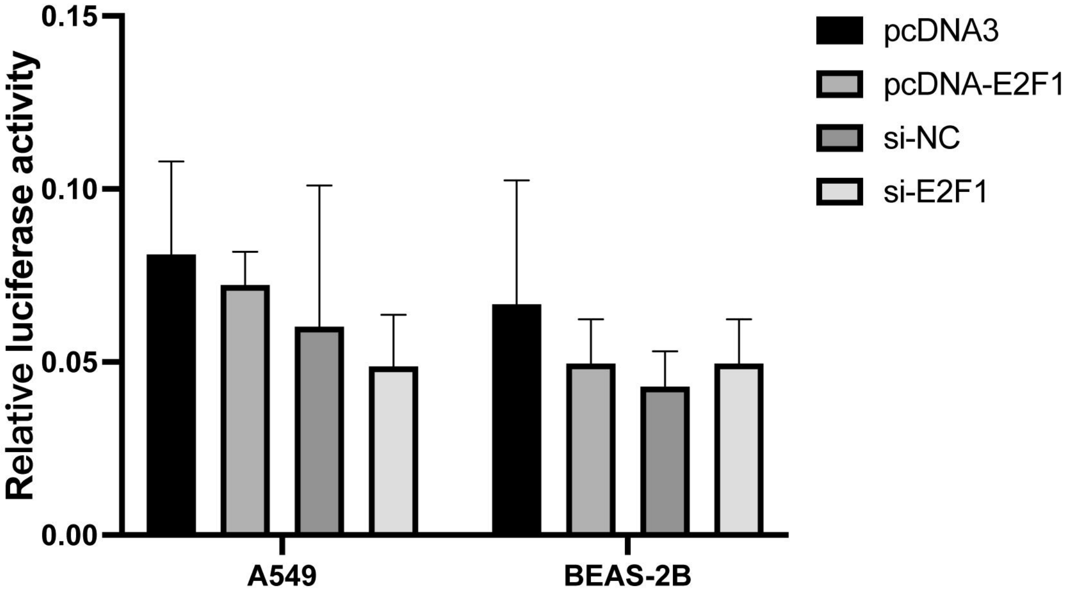

Figure 1 The change of E2F1 level to the activity of the pGL3-basic empty vector. The pGL3-basic empty vector were co-transfected with pcDNA3.1 (100ng), pcDNA-E2F1 (100ng), si-NC (100nM) or si-E2F1(100nM) into A549 and BEAS-2B cells. Luciferase activity was detected 24 h following transfection (*P*>0.05).
